# Model-Based Analysis on the Extractability of Information from Data in Dynamic Fed-Batch Experiments

**DOI:** 10.1002/btpr.1649

**Published:** 2013-01-23

**Authors:** Patrick Wechselberger, Patrick Sagmeister, Christoph Herwig

**Affiliations:** Institute of Chemical Engineering, Research Area Biochemical Engineering, Vienna University of TechnologyVienna, Austria

**Keywords:** data exploitation, bioprocess model, bioprocess optimization, dynamic experiments, quality by design

## Abstract

Dynamic changes of physiological bioprocess parameters, e.g. a change in the specific growth rate μ, are frequently observed during industrial manufacturing as well as bioprocess development. A quantitative description of these variations is of great interest, since it can bring elucidation to the physiological state of the culture. The goal of this contribution was to show limitations and issues for the calculation of rates with regard to temporal resolution for dynamic fed-batch experiments. The impact of measurement errors, temporal resolution and the physiological activity on the signal to noise ratio (SNR) of the calculated rates was evaluated using an in-silico approach. To make use of that in practice, a generally applicable rule of thumb equation for the estimation of the SNR of specific rates was presented. The SNR calculated by this rule of thumb equation helps with definition of sampling intervals and making a decision whether an observed change is statistically significant or should be attributed to random error. Furthermore, a generic reconciliation approach to remove random as well as systematic error from data was presented. This reconciliation technique requires only little prior knowledge. The validity of the proposed tools was checked with real data from a fed-batch culture of E. coli with dynamic variations due to feed profile. © 2013 American Institute of Chemical Engineers Biotechnol. Prog., 2013

## Introduction

### Motivation

The introduction of quality by design (QbD) is a driver for structured process development strategies based on sound science rather than empiricism. A main objective is the development of process understanding, both for the communication to the regulatory authorities as well as for business driven optimization efforts. Following the QbD initiative, pharmaceutical development is required to show in-depth understanding of factors with impact on product quality and their interactions.^1^–^3^ Increased process understanding can be acquired by proper experimental design (e.g., design of experiments; DoE).^4^ However, experiments generate huge amounts of experimental data besides CCPs (critical process parameters) and CQAs (critical quality attributes) as defined in the ISPE document,^3^ which can also contribute to process understanding. Multivariate data analysis provides numerous so called empirical or data driven tools to explore, structure, and classify data (e.g., PCA, artificial neural networks, cluster analysis etc. and also to explore correlations and dependency between variables (e.g., multiple linear regression, PCR/PLS-R, etc.).^5^–^8^ These can be very helpful tools; however, an important requirement for empiric models is representative data for the model problem, avoiding extrapolation from the training data set, which can potentially lead to misleading conclusions. Furthermore, when applied to a bioprocess, coefficients, and mathematical relations typically have no direct physiological meaning, hence interpretation and generation of knowledge from these is not straight forward.

A more direct approach to generate knowledge is using mechanistic models, which describe the system in question by fundamental knowledge (e.g., chemical or physical principles) of the interaction between process variables.^9^, ^10^ The advantage of mechanistic models is also a drawback; detailed knowledge of the process is not always available. In biological processes setting up mechanistic models is especially challenging due to the great complexity of the living cell. Looking into the cell this can be achieved e.g., by metabolomics or transcriptomics.^11^–^14^ However, in industrial process development time is a limiting factor; hence a simpler and faster approach is required. Valuable information on the cell physiology can be also acquired using a black box, unsegregated model of the cell^15^ where conversion rate for reactants and products (e.g., substrates: C-source, oxygen, products: biomass, carbon dioxide) entering and leaving the cell envelope (catalyst) are analyzed. Furthermore, specific rates and yields calculated from conversion rates, e.g., the specific growth rate μ, can be used for the description of the cell state.

### Time resolved quantitative data processing as a tool for pharmaceutical upstream process development

Because of the inherent complexity of the biological systems, process development for biopharmaceutical is a time- and labor extensive task. The available toolset for economic process development along QbD principles includes DoE, risk based factor reduction^16^, ^17^ as well as parallel processing^18^ and automation strategies.^19^, ^20^ The plethora of available on-line and offline analytical devices poses great opportunities for a fast progress in system understanding within process development.^21^, ^22^ However, strategies are needed to tie the individual measurements together in order to get a full picture of the bioprocess. Here, an approach based on general mass balances is used to transfer and condense the available on- and offline data into scale independent, time resolved information in the form of rates and yields. Rates and yields can be further processed via elemental balancing and reconciliation procedures,^23^ enhancing the trust in the obtained information. This constitutes an exploratory strategy for biopharmaceutical process development that can help understanding the physiological impact of process parameters on the system under investigation. Furthermore, specific rates and yields can be analyzed for defined time intervals and used for the detection of transient changes in the cell state induced by e.g., fluctuating process parameters.^15^, ^24^ Comparison of specific rates and yields can lead to valuable information to allocate a change in the physiological cell state, which can also relate to product quality.^25^, ^26^ Quantitative data processing lays the basis for the calculation of time-resolved specific rates and yield coefficients.

### Black box model/rate based quantitative process description

The typical microbial fed-batch process in red biotechnology has oxidative growth stoichiometry without primary metabolite formation (or the amount is too small to be considered). The applied black-box description reduces the complexity of the biological activity to a single stoichiometric formula: Substrate reacts with oxygen and the available nitrogen source forming carbon dioxide and biomass (Eq. 1). In industrial processes acetate productions is often avoided, either by use of glycerol, limiting substrate flux or strain selection. So, Eq. 1 is applicable to a broad range of industrial processes. However, this does not limit the approach, since Eq. 1 can be easily updated to consider acetate or other metabolites. In a process development environment, oxygen-, carbon dioxide fluxes and in substrate-limited cultures, such as fed-batch processes, the substrate flux, are typically measured in real-time. Biomass is typically measured offline. Applying elemental balances this general stoichiometric equation can be split into a subset of reactions that can be utilized for the calculation of reaction rates using a matrix formulation.^27^ Hence, conversion rates are accessible based on the data typically recorded in bioprocess development. Conversion rates can be easily processed into physiological information in the form of specific rates and yields. To extract time-resolved information, (specific-) rates and yields can be calculated for a finite time window, e.g., instantaneously between every measurement point or between two points separated by a discrete time interval. This time resolved rate calculation can be utilized for the detection of transient changes in bioprocesses.

**Equation for oxidative growth**


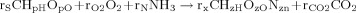
(1)

### Error propagation, signal quality, and noise reduction

Given available analytics, the calculation of specific rates and yields is straightforward and even feasible in real-time.^28^ However, these physiological meaningful process descriptors are composites of multiple measurements, each prone to random errors, drifts or even gross errors. Therefore, the extractable information needs to be differentiated from random noise, as already discussed for batch processes.^29^ Within this publication we expand the discussion of the rate-based bioprocess characterization approach to the fed-batch mode and induced conditions, proposing approaches for a scientifically sound quantification practice with respect to expected errors and expected biological variation, with special emphasis on the detection of transient changes of the metabolic state described by process variables such as rates and yields.

Next to the propagation of random measurement errors to specific rates and yields, gross errors such as sensor miscalibration and sensor drifts can distort their extractability from the available data. This especially accounts for the extractability of information with dynamically changing physiological conditions, e.g., the detection of a change in the specific growth rate μ or a change in biomass to substrate yield. Hence, the level of noise on such variables, which is called signal quality further on, has to be evaluated and set in context with quantitative measures. For this purpose the signal-to-noise ratio was defined as quality attribute for rate based evaluation of bioprocesses. The signal to-noise ratio (SNR) is a commonly used measure for data quality and can be used to assess the probability whether variations in the observed signal are physiological information and not random noise.

Averaging over a time window is a frequently used method to reduce random noise, since it is very easy to understand and to apply. In digital signal processing this is called moving average.^30^ However, there is a trade-off between gain in SNR ratio and the temporal resolution, for example required for tight process control, which needs elucidation for data evaluation in fed-batch processes. Less SNR ratio can be compensated by larger averaging intervals to remove random noise. Knowledge on the SNR to be expected, based on the errors on the participating measurements and the biological characteristics of the process, is useful in experimental planning. This allows an estimation of the maximum temporal resolution for the detectability of dynamic changes prior to experimentation, hence helps with definition of sampling intervals. For this purpose, this contribution utilizes an, *in silico* strategy, verified with real data, to analyze the propagation of measurement errors, averaging window size and physiological activity on the SNR of specific rates and yields. The goal is to tailor quantitative data processing to predefined objectives, expected errors and the system under investigation, aiming at a maximized time resolution while maintaining an objective dependent level of signal to noise.

Reduction of noise by a moving average method comes at the cost of temporal resolution as discussed above. Beyond averaging there are methods which introduce prior knowledge (e.g., process models) to remove noise instead. However, prior knowledge is not always applicable; hence there is a need for methods, which avoid making extensive use of such knowledge. Generally, applicable constraints such as elemental balances can remove measurement error by using very little prior knowledge.^28^

The methods presented in this contribution were developed using *in silico* data, since this allows for quick and easy generation of any kind of physiological variation and also adding artificial levels of random noise. Subsequently, the obtained results were validated and checked for their applicability using real fed-batch process data obtained from a recombinant process with dynamic changes in physiological variables.

### Goals

- We want to show limitations and issues for the calculation of rates with regard to temporal resolution for dynamic fed-batch experiments.
- We want to propose a real-time capable method for evaluation of dynamic variations due to physiological state in rate-based bioprocess quantification. A quantitative measure for signal quality was defined (SNR) and a rule of thumb equation is presented to estimate the SNR to get an idea on the ability to quantify expected physiological variations and to help with definition of sampling intervals beforehand.
- We want to present data reconciliation as a method for the reduction of measurement error using very little prior knowledge, while maintaining a high temporal resolution.
- The approach and the performance was investigated using data from a real fed-batch with dynamic variations in the data.

## Materials and Methods

### Culture

A recombinant K12 *E. coli* strain was used for the verification runs with stoichiometrically defined media. A shaking flask preculture (100 ml for inoculation of 6 l batch medium, in 1 l shaking flask with baffles) was inoculated from frozen stocks. After 8 h at 35°C, 180 rpm in the shaker the preculture was used to inoculate the bioreactor. Culture conditions were pH = 7, temperature = 35°C and DO_2_ > 20%. Using a glycerol concentration of 20 g/l the batch was finished within about 12 h. The end of the batch was detected by a drastic drop in the CO_2_ off-gas signal and an increase in dissolved oxygen (DO_2_). At this point an exponential fed-batch was initiated Eqs. 2 and 3 were used to calculate the feed profile for the exponential fed-batch. The specific growth rate before induction was set prior to the experiment, while constants such as the feed concentration (*S*_0_), density (ρ_feed_), initial biomass concentration *X*_0_ and initial volume *V*_0_ were measured. The biomass yield (*Y*_x/s_) was determined in prior experiments. The molecular weight of substrate and biomass (*M*_S_, *M*_X_) can be found in the literature or measured by an elemental analyzer. To generate variation in the specific growth rate, a linear feed was adjusted equal to the last feedrate of the exponential feed-profile, adjusted by a drop factor resulting in abrupt drop of the feedrate. This resulted in a reduced initial specific growth rate, e.g., from (=k) = 0.15 h^−1^ in the exponential phase to an initial μ = 0.1 h^−1^ in the linear phase.

**Feedrate in exponential fed-batch**


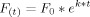
(2)

**Initial feedrate in exponential fed-batch**


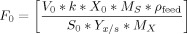
(3)

### Bioreactor setup and on-line analytics

#### Bioreactor

Two stainless steel bioreactors with working volumes of 10 and 20 l were used (Infors, Bottmingen, Switzerland). The systems come with a controller unit, which was used to adjust the process parameters: pH, temperature, aeration, reactor pressure, and stirrer speed. DO_2_ was controlled >20% using a step controller with reactor pressure, stirrer speed, and air flow as manipulated variable. The pH was controlled using an integrated digital peristaltic pump and NH_4_OH as a base. Air was filtered by a membrane-type filter and dispensed by a ring sparger. The culture vessel was sterilized at 121°C for 20 min by *in situ* steam sterilization prior to inoculation.

#### Off-Gas Analysis

CO_2_ and O_2_ in the off-gas were quantified by a gas analyzer (Servomex, UK; M. Müller AG, Switzerland), using infrared and paramagnetic principle, respectively. Air flow was quantified by a mass flow controller (Vögtlin, Aesch, Switzerland).

#### In-Line Capacitance Analysis

An annular type probe (Aber Instruments, Aberystwyth, Wales, UK) was used to measure capacitance during the fermentation. Capacitance values are calculated in real-time from the difference between two frequencies. At 1 MHz *E. coli* cells contribute to the capacitance while 10 MHz is the “background” depending on the medium, according to definitions of the supplier. The difference in capacitance relates to the viable cell concentration or more directly to intact biovolume, as only intact cells act as a capacitor.^31^

#### Data Management

For recording of process data the process information management system Lucullus from Biospectra (Schlieren, Switzerland) was used. This system was also used for closed loop control (feed bottle on balance).

### Quantitative evaluation of bioprocess data

#### Conversion Rates

Assuming oxidative metabolism, the bioreaction can be described by a single stoichiometric equation. Although there are many different chemical reactions running in parallel in living cells, the conversion rates in Eq. 1 (see section Black box model/rate based quantitative process description) represents the overall summarized effect of all the different reactions.

The conversion rates in Eq. 1 for the species substrate (*S*), biomass (*X*), carbon dioxide (CO_2_), ammonia (N) as well as oxygen (O_2_) in fed-batch mode can be calculated as follows:

**Conversion rate for substrate**


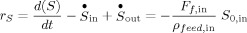
(4)

In fed-batch mode the outflow term 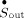
is zero and the accumulation term 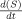
can be neglected, as long μ < μ_max_ hence the conversion rate *r*_s_ is only dependent on the inflow term 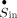, which is calculated from the feed rate.

**Conversion rate for biomass**


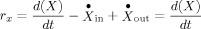
(5)

Since, there is no in- and outflow term *r*_x_ is equal to the accumulation term 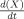. The biomass composition (CH_1.8_O_0.56_N_0.23_, ash: 5.5%) was determined experimentally (2400 CHN Elemental Analyzer, Perkin Elmer, Microanalytical Laboratory, University Vienna).

**Conversion rate for carbon dioxide**


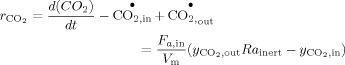
(6)

**Conversion rate for oxygen**


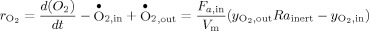
(7)

**Inert gas ratio**


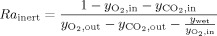
(8)

Because of the low solubility of O_2_ in the fermentation broth, 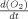
can be neglected. The term 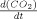
can be also neglected, since the solubility of CO_2_ in the fermentation broth is a mainly a function of temperature and pH, which are typically kept constant. Hence, the rates *r*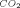 and *r*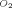 are dependent on the in- and outflow terms (Eqs. 6 and 7). *F*_a,in_, *y*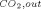, and *y*, out 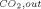 are measured, while *Ra*_inert_ (Eq. 8) depends on the dilution by water stripping describes the ratio between the in- and outflow term. *y*_wet_ is the off-gas concentration of O_2_ without bio-reaction and relates to the dilution by water stripping.^32^

The mass of the culture broth during the fed-batch was calculated by a general mass balance (Eq. 9). This balance includes ingoing and outgoing liquids (*F*_f,in_, *F*_b,in_), gases (*r*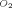, *r*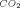), water stripping (*S*_water_, calculated from *y*_wet_) and the sampling rate (*f*_sample_).

**General mass balance**


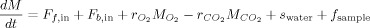
(9)

#### Specific rates and Yields

Conversion rates are the basis for the computation of yields (Eq. 10), which are defined as ratios between rates (e.g., biomass per substrate). Specific rates (Eq. 11) are typically conversion rates related to the biomass.


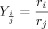
(10)


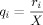
(11)

#### Constraints

**General form of constraints**


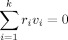
(12)

Using the law of conservation, elemental balances can be imposed on the every element of the bio reaction as constraints (Eq. 12). In which *r* is the rate vector and *v* is the vector of coefficients for each element. This is useful as a consistency check of the data and to calculate nonmeasured items. In this contribution two balances were used, the carbon (C) balance and the degree of reduction (DoR) balance.^29^

#### Consistency Check

A statistical test adapted from the Ref.^33^ was applied to get a quantitative measure on integrity of the observed system, based on the elemental balances imposed in section “Constraints.” Equation 12 can be written in matrix form (Eq. 13):

**Matrix form of constraints**


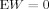
(13)

*W* is the vector of the measured volumetric rates *r* and *E* is the elemental matrix with the coefficients *v*.

For noisy data a residue vector ε is added (Eq. 14):

**Matrix form of constraints with residue vector**


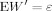
(14)

For each rate an expected error (by default 3% error on each rate) is specified in the variance-covariance matrix ψ of the rates and is assumed to be noncorrelated (square with the errors for each rate in the diagonal). The result of the statistical test value *h* is calculated with ϕ as the variance-covariance matrix of the residuals Eqs. 15 and 16. The hypothesis of not having any errors exceeding the expected error specified in ψ is rejected if *h* is greater than a certain threshold value. This threshold value can be read from χ^2^ distribution, which depends on the degree of redundancy of the equation system (or also the degree of freedom of the χ^2^ distribution) and the significance level α (by default 0.9). The default α degree of redundancy of one (= estimation of one rate) or two (= no estimation, only consistency check) results in a threshold of 2.71 or 4.61 for the statistical test value, which is exceeded if the current error is higher than the expected error. In Ref. 29, the expected error was assumed to be 3% error on each rate. As shown in the Ref.^29^ less than 3% error on each rate (the variance-covariance matrix ψ has 0.03 in the diagonal) is feasible if the averaging window (Δ*t*) is chosen accordingly (>2 h). An error of 3% on each rate results in a deviation of about 10% on the C- and DoR balance, which is also the assumed cumulative error on all rates. The degree of redundancy of the equation system is equal to the rank of *E* if no conversion rates are estimated or to the rank of *R* if conversion rates are estimated.

**Variance-covariance matrix**


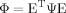
(15)

**Statistical test value**


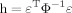
(16)

#### Data Reconciliation

A data reconciliation procedure according to the Ref.^23^ was applied. In addition to estimation of nonmeasured conversion rates, redundancy in the equation system can also be used to adjust the conversion rates to simultaneously close all elemental balances imposed in section “Constraints”. The lumped residues of the equation system are distributed along the rates according the expected error for each rate. Using a least squares approach the goal of reconciliation is to find a measurement error vector δ to calculate the reconciled vector *W*_b_ (Eq. 17), hence the vector of the best estimates of the volumetric reaction rates to fit all constraints. The solution to this problem is adapted from the Ref.^34^ (Eq. 18).

**Calculation of the reconciled vector***W*_b_


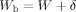
(17)

**Calculation of the measurement error vector δ**


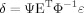
(18)

### *In silico* data generation

An *in silico* data set was generated using Excel (Microsoft, Redmond, USA) according to the equations in section “Quantitative evaluation of bioprocess data.” Normally distributed random noise (F-distributed) was added to this data to evaluate extractability of information based on the signal to noise ratio (see section “Calculation of rates by finite difference approximation”).

### Calculation of rates by finite difference approximation

Since, there is no way to directly measure the conversion rate for some of the species in the bioreaction (Eq. 1), these have to be calculated from measurements at discrete time points, e.g., the biomass conversion rate. A rate can be calculated from time-value pairs by numeric differentiation using simple finite difference approximation according to Eq. 19. While Δ*i* corresponds to difference from one measurement of the species (e.g., biomass) to the other, Δ*t* is defined by the sampling interval, or multiples of it.

**Finite difference approximation for calculation of conversion rates**


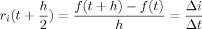
(19)

### Calculation of statistical parameters

The standard error of the arithmetic mean (Eq. 20) is the standard deviation(s) of the arithmetic mean *x* with multiple replicates (*n* replicates). Replicates improve the estimation and result in a smaller standard error.^35^ The SNR (Eq. 21) compares the arithmetic mean (*x*) of a signal to the level of the background noise or the standard deviation of the signal(s). The limit of detection and quantification (Eqs. 22 and 23) are terms known from the validation of methods in analytical chemistry and can be used as thresholds for SNR for the goals detection or quantification of a component or, in this contribution, variations of specific rates, and yields.

**Standard Error**


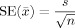
(20)

**Signal to noise ratio**


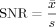
(21)

**Limit of detection**


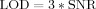
(22)

**Limit of quantification**


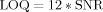
(23)

## Results and Discussion

### Error propagation in fed-batch

One of the goals of this contribution is the evaluation of the extractability of information by quantitative analysis of typical data from a bioprocess; hence error propagation from raw data has to be analyzed. Table 1 shows typical measurement errors (according to suppliers' specification) for on-line devices and also for biomass quantification. The latter is typically much higher than all other items. For off-line biomass quantification this error can be reduced by replicates according to the equation for the standard error of the arithmetic mean (Eq. 20). For example using four replicates the expected relative error is reduced from 4% to 2%. Obviously more replicates come with diminishing effects and also time consuming extra work. Typically probes for in-line quantification of biomass come with similar or even higher relative errors.

**Table 1:** Methods, Standard Deviations, Relative Error (Biomass) and Error Types Typically for Methods/Devices Typically used for Quantitative Evaluation of Fed-Batches

Device/Method	Relative Error	Type of Error	Range	Unit
Feed balance	1	Absolute error	0–35.000	(g)
Base balance	1	Absolute error	0–35.000	(g)
Reactor balance	1	Absolute error	0–35.000	(g)
O_2_ off-gas analysis paramagnetic	0.02	Relative error	0–26	(%)
CO_2_ off-gas analysis infrared	0.01	0.1% absolute error on full scale 0–10%	0–10	(%)
MFC_Air thermal mass flow meter	0.035	Relative error	0–40	(l/min)
Biomass quantification e.g.: dryweight, capacitance	2% (dry weight, for *s* = 4 %) and 4 replicates according to Equation 20), or 8% (capacitance)	Relative error	>0.1	(g/l)

**Figure 1:**
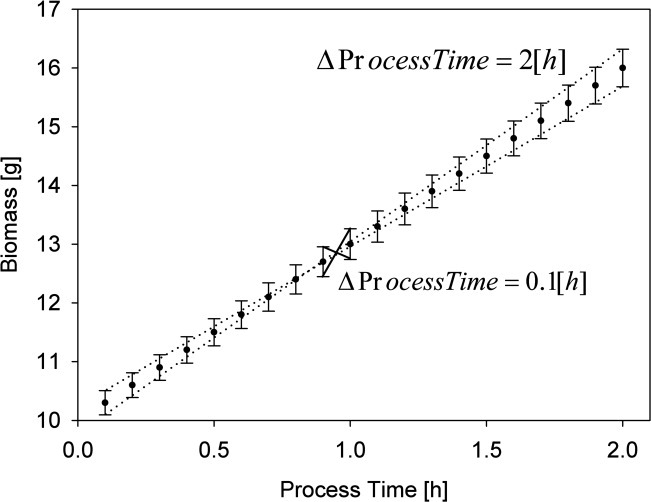
Graphical representation for the numeric differentiation of biomass growth using a small Δ*t* (solid line, 0.1 h of process time on the *x*-axis) and a large Δ*t* (dotted line, 2 h of process time on the *x*-axis); 2% relative standard deviation on biomass measurements (*y*-axis).

Using finite difference approximation according to section “Calculation of rates by finite difference approximation,” it is typically recommended to choose Δ*t* as small as possible; however, error propagation e.g., from biomass measurements is highly unfavorable, so smaller Δ*t* (further on also called averaging window) leads to more noise on the calculated rate (see Figure 1). Furthermore, the specific growth rate directly increases the signal to be evaluated (Δ*i*), since most of the other rates are directly proportional to it. In a previous contribution^29^ it was shown that, summing up, SNR is dependent on the following factors: the biological activity, the averaging window (or temporal resolution), and the measurement error. With a greater signal and lower measurement error, higher time resolution can be achieved with sufficient signal quality.^29^ Connecting two samples for biomass in Figure 1 by a line, is in fact the graphical representation for the calculation of the biomass conversion rate by finite difference approximation according to section “Calculation of rates by finite difference approximation.” Random error is considered as presented by the error bars. Looking at Figure 1 it is pretty obvious that the resulting rate is much more governed by random error (here = 2% relative error on each sample) if Δ*t* is small (solid line, 0.1 h on the *x*-axis) compared to larger Δ*t* (dotted line, 2 h on the *x*-axis), since the connecting lines (= graphical representation for the calculation of the biomass conversion rate) differ much more in the first case due to random error. This is even though the actual rate is constant over the whole range, since a linear growth function was used to generate the data points. Other growth functions such as exponential growth lead to similar results (not shown). Filtering techniques, which can be used to smooth rates, typically also come at the cost of temporal resolution (e.g: moving average filter), or require prior knowledge (e.g: a process model).

**Figure 2:**
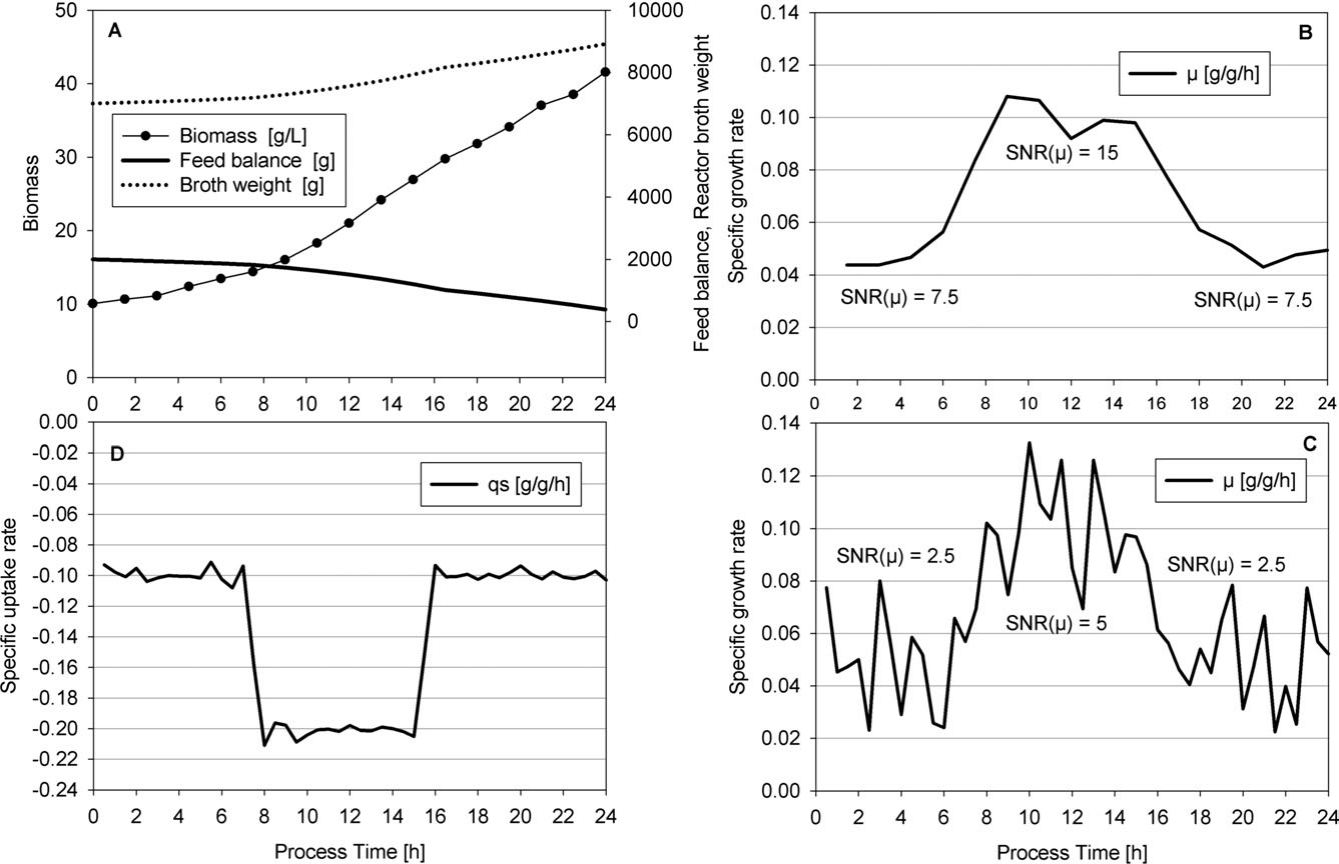
*In silico* generated data for a typical fed-batch (Microbial culture, Glucose Feed 400 g/l) with an artificial increase from μ = 0.05 h^−1^ to μ = 0.1 h^−1^ at process time = 8 h and back to μ = 0.05 h^−1^ at process time = 16 h (A) Progression of raw data required to calculate specific rates: biomass concentration (artificially added noise: 1.5% relative error), feed balance and reactor broth weight; (B) Specific growth rate with a Δ*t* of 3 h according to Eq. 19 with a signal-to-noise ratio of 15 and 7.5 at μ = 0.1 h^−1^ and μ = 0.05 h^−1^, respectively; (C) Specific growth rate with a Δ*t* of 1 h according to Eq. 19 with a signal-to-noise ratio of 5 and 2.5 at μ = 0.1 h^−1^ and μ = 0.05 h^−1^, respectively; (D) Specific uptake rate with a Δ*t* of 1 h as used in Eq. 19.

While this error propagation is easily understood for the example discussed above (also see a previous publication^29^), things get more complex if dynamic variations due to cell metabolism are added, e.g., due to feed profile. In fact, we want to differentiate those variations from random noise. Figure 2A shows *in silico* generated data from a typical microbial fed-batch, which are required to calculate specific growth rates: the biomass concentration, the reactor broth weight and the weight of feed over time. Noise according to Table 1 was artificially added. A variation in the specific growth rates from μ = 0.05 h^−1^ to μ = 0.1 h^−1^ at process time = 8 h and back to μ = 0.05 h^−1^ at process time = 16 h was simulated, which is barely noticeable in the raw data (Figure 2A). Figure 2B shows specific rates calculated from the raw data in Figure 2A with a Δ*t* of 3 h according to Eq. 19. Figure 2C shows specific rates calculated from the same raw data, but with a Δ*t* of 1 h according to Eq. 19. A relative error for biomass quantification of 1.5% with a Δ*t* of 1 h leads to variations of up to about as large as the signal (the specific growth rate) itself, as seen in Figure 2C, which makes visual interpretation of this plot very difficult. In Figure 2B visual interpretation is much easier, due to the Δ*t* of 3 h according to Eq. 19. The SNR can be used to evaluate the quality of the calculated specific growth rate on a quantitative basis. Since, the noise on the signal is known and constant for a defined time window in this artificially generated example, calculation of standard deviation and arithmetic mean to get the SNR according to Eq. 21 is straight forward. A signal to noise ratio of 3 (= signal is 3 times than the residual standard deviation) is defined as the limit of detection while a ratio of 12 is the limit of quantification.^36^ With a SNR of 12 a 100% variation of the signal can be reliably detected, to quantify a smaller variation the SNR should be even higher (e.g., a SNR of 120 for 10%). Accordingly, it is hardly possible to extract useful information in Figure 2C, since the signal to noise ratio is barely good enough to detect a change (SNR = 2.5 or 5). The window should be increased to 3 h or higher, to get a specific growth rate with a SNR higher than three or preferably >12 (Figure 2B), in order to be able to distinguish between random noise and real physiological variability based on previously established definitions for limit of detection and quantification. Obviously, the SNR increases linearly with the specific growth rate (μ), since μ is in the top of the fraction in Eq. 21. Figure 2D shows the specific uptake rate (*q*_s_) for this data set. Since, *q*_s_ was calculated using data from the feed balance, which comes with a much lower measurement error as compared to the biomass measurement, the resulting rate is less noisy. If the sampling strategy and the process setup are optimized to meet signal quality requirements for the most noisy process variable (here the biomass concentration), all other process variables will follow the requirements as well.

**Figure 3:**
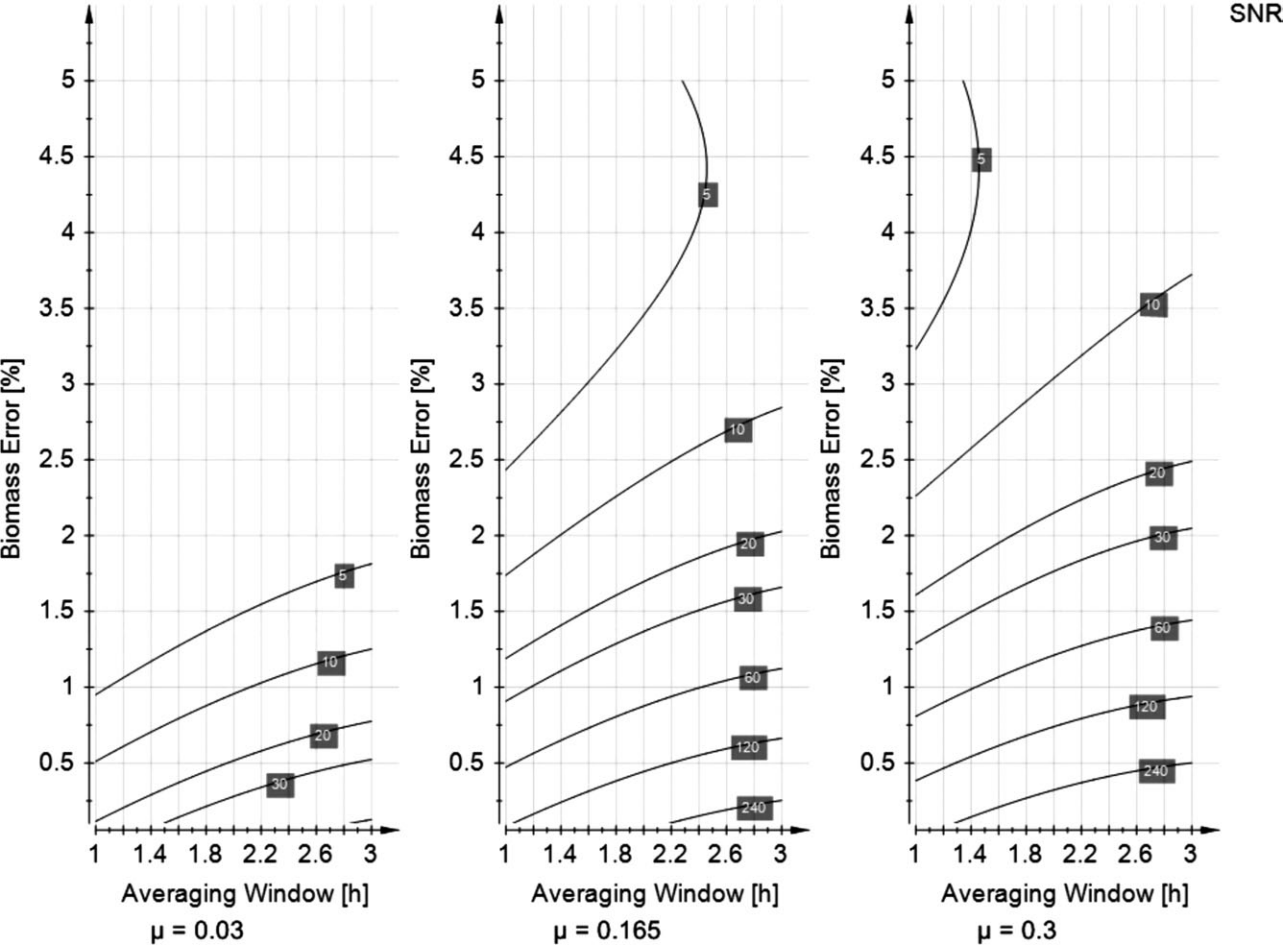
Relation of biomass error, averaging window and specific growth rate (*x*-, *y*-, and *z*-axis) to signal to noise ratio (isolines with labels).

The dependency of SNR on the specific growth rate h^−1^, averaging window h (Δ*t* as used in Eq. 19) and biomass error % on a broader range is shown in a contour plot in Figure 3. The plot was generated by setting up a multi-linear regression model (Software: Modde, Umetrics, Sweden). Noisy (biomass) data result in a low SNR, especially at low growth rates (e.g., μ = 0.03 h^−1^). This can be alleviated by either using a higher averaging window (Δ*t* as used in Eq. 19) at the cost of time resolution or more replicates for the biomass quantification. However, this is not always applicable e.g., with real time measurement by a capacitance probe, since additional measurements by increasing the sampling frequency are no real replicates.

**Figure 4:**
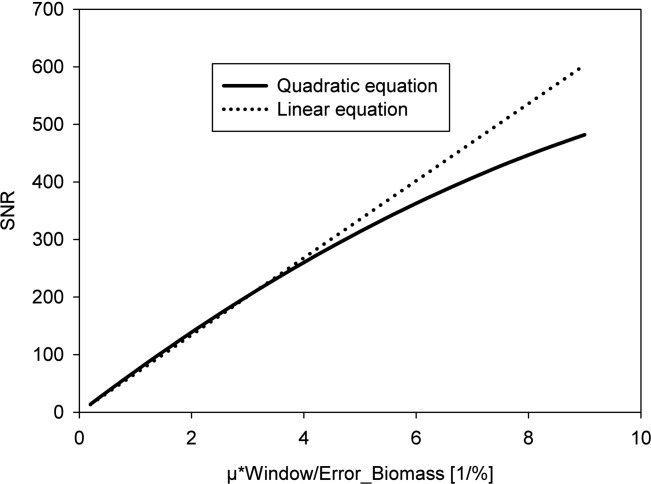
Relation of a combination of biomass error, averaging window (Δ*t*) and specific growth rate to signal-to-noise ratio (SNR); Comparison between linear and quadratic equation.

The model can be condensed in one coefficient, by putting the positive effects (specific growth rate and averaging window Δ*t* as used in Eq. 19) in the top and the negative effects (biomass error) in the bottom of the fraction (Eq. 24). This also represents the signal in relation to the error in analogy to general equation for SNR (Eq. 21). As shown in Figure 4 the model has a quadratic effect for higher SNRs, but can be approximated linearly at lower SNRs (Eq. 24). The quadratic effect is due to the finite difference approximation according to Eq. 19; too high values for h are counterproductive.

**Rule of thumb equation for SNR**


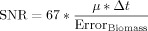
(24)

### Noise reduction using little prior knowledge: reconciliation

Higher averaging windows (Δ*t* as used in Eq. 19) can only deal with random noise; systematic errors cannot be reduced this way. A procedure according to a previous publication^23^ can be used to reconcile rates to remove random error and even more importantly also small systematic errors such as slight miscalibration of equipment, instrument drifts, and even minorly aberrant constants (e.g., feed concentration). The basic idea is to adjust the rates to fit constraints (elemental balances) according the expected error (e.g., according to manufacturer specifications or method replicate error) on each rate. This error has to be specified in the variance-covariance matrix ψ. As long the constraints were based on correct assumptions (e.g., stoichiometric equation) and the experimental errors do not exceed the errors specified in ψ, random and also systematic error can be effectively removed by reconciliation.^23^ However, the specified errors have to reasonably substantiated (e.g., according to manufacturer specifications or method replicate error), else the reconciliation procedure may result in artifacts. Furthermore, the χ^2^ distribution (used for the definition of the threshold value for the *h*-value, see section “Consistency check”) is for normally distributed values. Systematic error does not necessarily follow a normal distribution (99% of the observed values are distributed within three standard deviations) and may be constant. Hence, the threshold for the *h*-value according to the χ^2^ distribution might be too forgiving if a major fraction of the residuals is due to systematic error. This should be considered if the error structure on the measurement is known.

**Figure 5:**
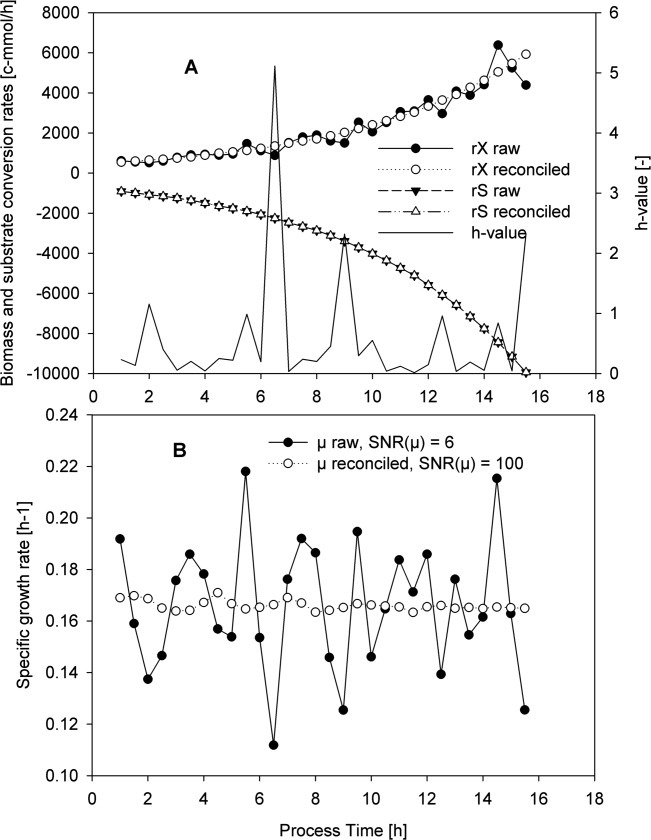
Rates calculated from in-silico generated fed-batch data (Microbial culture, Glucose Feed 400 g/l) Window for rate calculation (Δ*t*, Eq. 19): 1 h. Specific growth rate = 0.165 h^−1^; Biomass error = 2.55%; (A) Raw and reconciled conversion rates together with the corresponding h-value; (B) Specific growth rate (μ) from raw and reconciled data.

The biomass measurement is typically more prone to error as compared to other data, it can be expected that most of the noise is on this rate. A good estimate for the expected error is the reciprocal of the SNR, which can be simply calculated using Eq. 24 (which was inferred from Eq. 21). The second highest noise is on the rate for oxygen uptake, which is prone to systematic error; e.g., dilution by water in the off-gas, which can also vary during the process. The error on the other rates is mainly systematic as well (miscalibration, sensor-drift, measurement error on constants such as feed concentration etc.), since the random measurement error propagated by on-line devices (see Table 1) is typically negligibly small (<10^−4^ %). Assumptions for errors on necessary items for the calculation of rates are shown in Table 2 and based on that recommendation for ψ are given. Here, most of the systematic error is due to constants acquired by measurement (e.g., feed concentration, density, water dilution etc.); hence, it is safe to assume most of the systematic error is normally distributed. Figure 5A shows the biomass production rate (rX) for a typical mircobial fed-batch, which was reconciled according to section “Data reconciliation” using the errors specified in the variance-covariance matrix ψ from Table 2. The other rates (r_S_, rCO_2_, and rO_2_) were reconciled as well but as explained above most of the error is in the biomass rate. The *h*-value is a statistical test (threshold = 4.61, can be read from the χ^2^ distribution, dF = 2, α = 0.9), which states whether the residuals on the balance are within the expected range according to ψ. If the threshold is exceeded, the error is higher than previously specified. The reconciliation result might be still useful; however, the procedure fitted higher error to the elemental balances than previously expected. This can be also due to a wrong assumption of the growth stoichiometry, e.g., unaccounted formation of metabolites. As can be seen in Figure 5B the SNR (according to Eq. 21) is increased from 6 to 100 by reconciliation only. This means, due to the removal of measurement noise, a transient change in rates and yields almost 15 times smaller can be reliably detected. Or, if the physiological variation is expected to be very dynamic, the temporal resolution could be increased by a factor of 15, to detect short time variations enabling process control for example.

**Table 2:** Assumptions for Errors on Necessary Items for the Calculation of Rates and Recommendation for the Variance-Covariance Matrix ψ

Rate	Influencing Factors	Relative Error on Factor (%)	Effect to the Rate (%)	_ψ_
rx	Biomass quantification error	e.g.: 2	1/SNR × 100	1/SNR + 0.01
rx	DoR Biomass	e.g.: 1		
rs	Feed concentration	e.g.: 1		0.03
rs	Feed density	e.g.: 1		
rCO_2_		Miscalibration/sensor drift plus random error		0.01
rO_2_		Miscalibration/sensor drift plus random error		0.06
rO_2_	yo2_wet	0.2	up to 6	

### Verification with data from a real fed-batch

**Figure 6:**
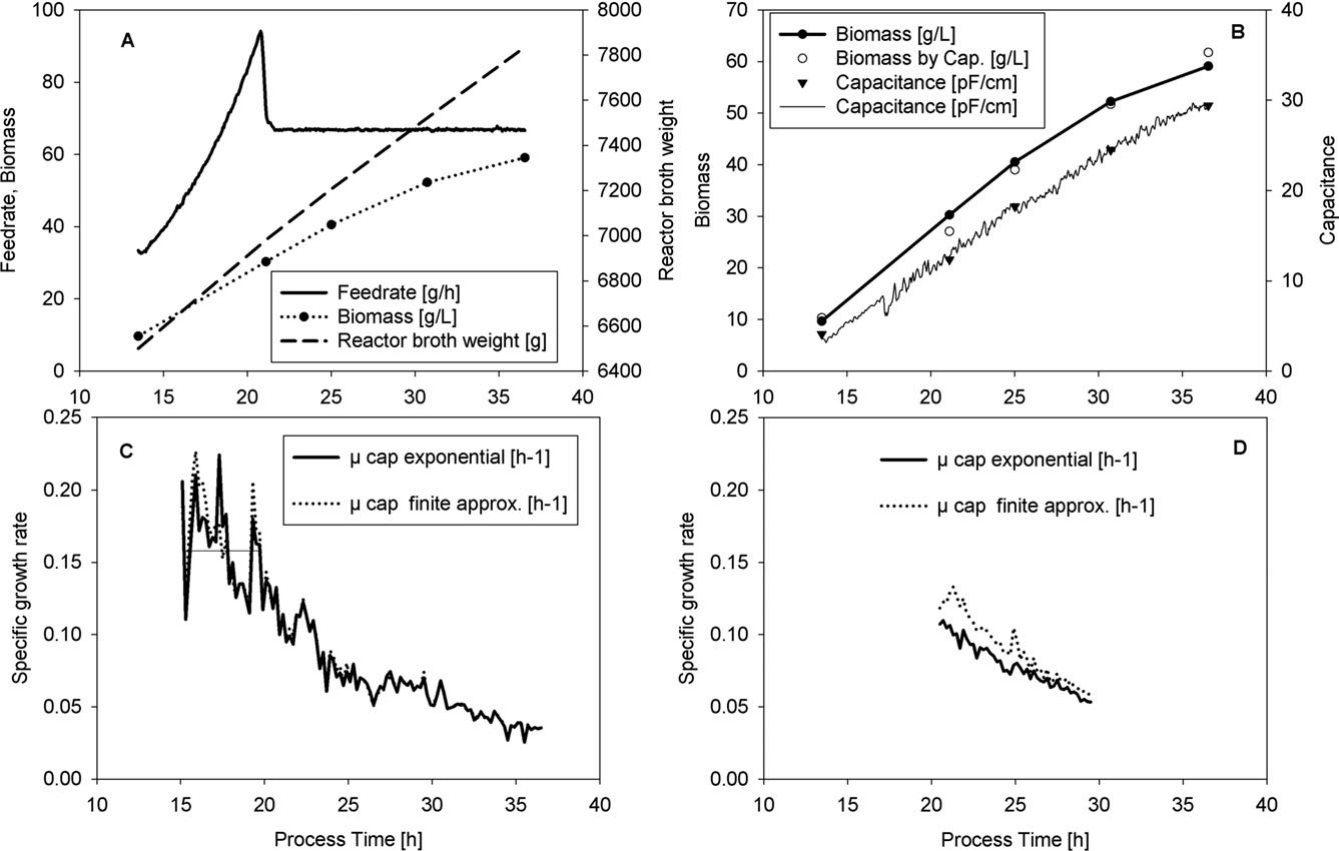
Data from an *E. coli* fed batch experiment; (A) Off-line biomass dry weight, feed-rate according to an exponential feed-profile from 13 to 22 h process time with a μ set of 0.15 h^−1^, followed by a linear feed phase with a μ-initial of 0.1 h^−1^ (as explained in section “Culture”) and reactor weight; (B) Linear regression of signals from the capacitance probe; (C) Calculation of the specific growth rate from biomass quantified by the capacitance probe using a Δ*t* of 4 h according to Eq. 19, which results in an SNR of three according to Eq. 21; (D) Calculation of the specific growth rate from the same biomass data quantified by the capacitance probe using a Δ*t* of 15 h according to Eq. 20, which results in an SNR of three according to Eq. 21. “μ cap exponential” and “finite difference approx.” were calculated by Eq. 25 and by Eq. 11 together with Eq. 19, respectively.

The approach was verified using real data from an *E. coli* fed batch. Following batch phase (data not shown) an exponential fed-batch with a μ_set of 0.15 h^−1^ was initiated, as shown in Figure 6A (process time 13 h). This was followed by a linear feeding phase with a μ_initial = 0.1 h^−1^ at process time 22 h. Because of the linear feedrate and the further increasing biomass, the specific growth rate decreased over time. The sampling interval was chosen according to Eq. 24. With a measurement error for biomass of 2% (Table 1) and an initial growth rate of 0.1 h^−1^ a Δ*t* (as used in Eq. 19) of 4 h is required to get a signal to noise ratio >12 (limit of quantification). This way a reasonable maximum sampling frequency was determined, since additional data points do not contribute as replicates, hence cannot reduce random noise.^29^ Furthermore, the presented approach was also applicable to signals from a biomass probe in the same experiment, a capacitance sensor with a very high sampling frequency compared to off-line biomass quantification (section “In-line capacitance analysis”). There was clearly a lot of random noise on the signal of the probe as can be seen in Figure 6B in addition to potential systematic error by measurement principle. The capacitance signal is dependent on electrical properties of the cells and can be related to intact bio volume or also to biomass dry weight. Linear regression analysis came up with a relative standard error of 8%, which results in a SNR of 3 with a Δ*t* of 4 h (Figure 6C) or an SNR of 12 with a Δ*t* 15 h as used in Eq. 19 (Figure 6D). While it is hardly possible to distinguish between the exponential phase and the linear phase in Figure 6C, this is impossible in Figure 6D. This clearly shows the limits of noise reduction by using bigger Δ*t* as used in Eq. 19. If the temporal resolution (15 h, which is in fact half of the fed-batch) is too poor, one might miss important process events. Furthermore, using a large Δ*t* such as 15 h as used in Eq. 19, the approximation error from finite difference approximation can have a significant impact on the calculated growth specific rate. To evaluate the impact of this approximation error, prior knowledge in the form of the function for exponential growth function (Eq. 25) was used instead of Eq. 11 together with Eq. 19, which is possible, since it safe to assume growth is exponential in the exponential phase.

**Calculation of μ the capacitance signal (*i* at time points *t*_1_ and *t*_2_)by exponential growth function**


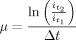
(25)

With a Δ*t* of 4 h (Figure 6C, μ cap exponential and finite difference approx.) there is hardly any difference between the specific growth μ calculated from the capacitance signal by finite approximation and the exponential growth function respectively, but with a Δ*t* 15 h there is major deviation in the growth rates for exponential phase, as shown in Figure 6D (μ cap exponential and finite difference approx.). The specific growth rate is artificially lowered by finite approximation. Summing up, a large Δ*t* of 15 h is probably not useful.

### Reconciliation of fed-batch data

**Figure 7:**
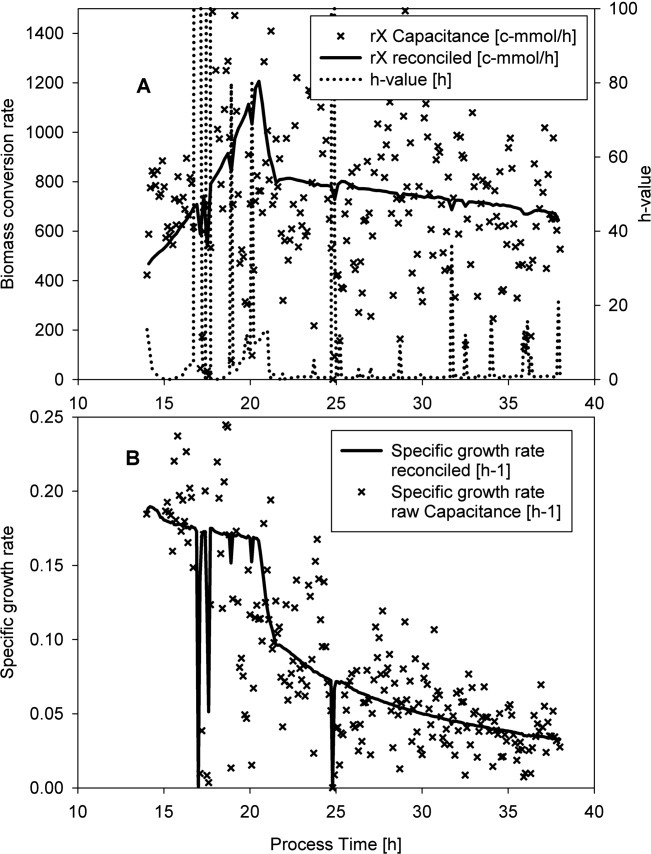
Comparison of the specific growth rates calculated from the raw capacitance signal and after the reconciliation procedure. The data is very scattered due to the high temporal resolution (Δ*t* = 1 h).

As discussed above a Δ*t* of 15 h is probably not useful, while a Δ*t* of 4 h results in a specific growth rate with an SNR of only 3, which is not satisfactory since this way, variations in the growth rate can only be detected but not quantified (following the definitions for limit of detection and quantification). Hence, we want to introduce prior knowledge in the form of elemental balances and reconcile the data (section “Data reconciliation”). Using this approach, the temporal resolution can be increased due to the effective removal of measurement error. Using the Capacitance data from section “Verification with data from a real fed-batch”, a Δ*t* of 1 h, as used in Eq. 19 results in a SNR of 0.84 according to Eq. 21, which means the random noise on the signal is greater than the signal itself. Hence, the specific growth rate in Figure 7(B) is more scattered (Δ*t* = 1 h), compared to Figure 6 (C, Δ*t* = 4 h). This clearly shows limited use of noisy signals such as the capacitance signal to calculate rates with a high temporal resolution. Nevertheless this high level of noise can be effectively removed by reconciliation (Figure 7A) as long the *h*-value is below the threshold value (4.61), which is true for most of the process. At process time 17 h there was a small problem with the off-gas analyzer (data not shown), while at process time 20 h the manipulation of the feed-rate controller disturbed the input rates for the reconciliation procedure, hence increased residuals on the elemental balances, which resulted in *h*-values above the threshold value (4.61). Figure 7(B) shows a comparison of specific growth rate calculated from the raw capacitance signal using a Δ*t* of 1 h and the specific growth rate after the reconciliation procedure. The reconciliation procedure was able to retrieve the μ profile from the rate calculated based on the capacitance signal (which was very scattered due to low Δ*t*); however, the capacitance signal did contribute very little to the result. Nevertheless, the reconciliation procedure allows making use of higher measurement frequencies, since less averaging time (Δ*t* as used in Eq. 19) is required to deal with noise.

## Conclusions

A methodology to assess the extractability of information from fed-batch experiment with varying specific growth rates was presented. The approach was verified with real data from an *E. coli* fed-batch. The presented approach applies to conversion rates calculated from discrete time-values pairs by finite difference approximation (section “Calculation of rates by finite difference approximation”). Removal of random noise by averaging (Δ*t* as used in Eq. 19) comes at the cost of temporal resolution. The SNR was established as a quantitative measure to evaluate the extractability of rate-based information (signal quality). Thresholds for the detection and quantification of dynamic variation in rates were established according to definitions known from analytical chemistry. This can be used to find the required amount of averaging (Δ*t* as used in Eq. 19) and to evaluate what level of variation can be detected in an experiment. Hence, a reasonable sampling frequency can be defined from these considerations, since sampling at a higher frequency will not bring additional benefit. Obviously, the dynamic of variation of the specific rates or yields of interest, has to be smaller than the previously defined Δ*t*; higher dynamic cannot be resolved by this approach unless the equipment or the methods are improved for a lower measurement error.

Other approaches can make use of prior knowledge to improve the SNR without loss of temporal resolution. The efficiency of reconciliation was shown; it can effectively remove random noise and systematic error by introducing elemental balances as constraints. Thus, the extractability of information was increased with very little effort. This way much smaller values of Δ*t*, as used in Eq. 19, are allowed to resolve more dynamic processes. Concomitantly a statistical test was presented which provides a good measure of the reliability of the result of the reconciliation procedure.

The results also suggest that calculation of the biomass conversion rate from off-gas signals, which are often available at high quality, is superior to calculation of the biomass conversion rate from noisy data from on-line probes such as the capacitance probe used in this contribution. This clearly shows limited use of noisy signals such as the capacitance signal to calculate rates with a high temporal resolution. However, the capacitance probe can add redundancy to the bioreactor monitoring system if high temporal resolution (low Δ*t*) is not important and allows for calculation of the total biomass in the bioreactor, which is required for specific rates (Eq. 11).

The methodology is a useful tool for successful experimental planning, therefore we want to propose a short “how to” guide.

### Stepwise guide to assess signal quality and extractability of information

- Define the specific growth rate and measurement error for biomass.
- Define expectations or requirements on the level of variations of the specific growth rate or yield.
- Define the required SNR, e.g., to quantify a 50% variation of the specific growth rate, an SNR of 2*12 = 24 is required.
- Use Eq. 24 to calculate the required Δ*t* (as used in Eq. 19); this will also define the temporal resolution.
- If the temporal resolution is not adequate for the problem, check if reconciliation is possible with the available analytics (are all items to calculate the rates required for the elemental balances available?) or evaluate the applied methods and equipment with regard to measurement error.

## Symbols

*t* = time (h)

I = any item

S = total amount of substrate in the cultivation broth (C mol)

= substrate feed rate (C mol/h)

*r* = conversion rate (C mol/h)

*q* = specific rate (g/g/h)

*Y* = Yield (C mol/C mol)

*F* = flow/feed rate (g/h) for liquid and (nl/h) for gas

*C* = concentration (C mol/l)

*X* = total amount of biomass in the cultivation broth (C mol)

*Y* = mole fraction (–)

*V*_m_ = molar volume of gas at norm condition (0°C and 1 atm) (nl/mol)

*Ra*_inert_ = inert gas ratio (–)

*y*_wet_ = O_2_ fraction in off-gas diluted by water content (without bioreaction) (–)

*N* = total amount of ammonium in the cultivation broth (mol)

O_2_ = total amount of oxygen in the cultivation broth (mol)

CO_2_ = total amount of carbon dioxide in the cultivation broth (mol)

*Z*_i_ = elemental composition of component *i* in biomass (–)

*p*_i_ = elemental composition of component *i* in substrate (–)

*V* = volume of the cultivation broth (l)

ex_H2O_ = water content in off-gas (–)

*M* = molecular weight (g/c mol)

ρ_feed_ = density of feed (g/l)

*S*_0_ = feed concentration (g/l)

γ = Degree of reduction (–)

ν = coefficients e.g.: ν (–)

OD = optical density 600 nm (–)

*k* = specific growth rate if used for feed rate calculations (h^−1^)

μ = measured specific growth rate (h^−1^)

*s* = standard deviation

*x* = arithmetic mean

Ψ = variance-covariance matrix

*h* = statistical test value

*S*_water_ = water stripping (g/h)

*f*_sample_ = sampling rate (g/h)

## Indices

in = input

out = output

conv. = conversion

acc. = accumulation

s = substrate

f = feed

x = biomass

a = air

CO_2_ = carbon dioxide

O_2_ = oxygen

N = ammonium

b = base

O = oxygen

H = hydrogen

n = nitrogen

m = measured

c = estimated

i = item number i

j = item number j

t = time point t

0 = initial
